# Challenges and Solutions for Commercial Scale Manufacturing of Allogeneic Pluripotent Stem Cell Products

**DOI:** 10.3390/bioengineering7020031

**Published:** 2020-03-28

**Authors:** Brian Lee, Breanna S. Borys, Michael S. Kallos, Carlos A. V. Rodrigues, Teresa P. Silva, Joaquim M. S. Cabral

**Affiliations:** 1 PBS Biotech, Inc., Camarillo, CA 93012, USA; 2 Department of Chemical and Petroleum Engineering, Schulich School of Engineering, University of Calgary, 2500 University Dr. NW, Calgary, AB T2N 1N4, Canada; bsborys@ucalgary.ca (B.S.B.); mskallos@ucalgary.ca (M.S.K.); 3 Ibb—Institute for Bioengineering and Biosciences and Department of Bioengineering, Instituto Superior Técnico, Universidade de Lisboa, Lisboa 1049-001, Portugal; carlos.rodrigues@tecnico.ulisboa.pt (C.A.V.R.); teresasilva@tecnico.ulisboa.pt (T.P.S.); joaquim.cabral@tecnico.ulisboa.pt (J.M.S.C.)

**Keywords:** allogeneic cell therapy, induced pluripotent stem cell, human embryonic stem cell, cell aggregate, expansion, differentiation, scalable manufacturing, scale up, single-use bioreactor, Vertical-Wheel, U-shaped vessel, computational fluid dynamics, shear stress, turbulent energy dissipation rates, homogeneous hydrodynamic environment

## Abstract

Allogeneic cell therapy products, such as therapeutic cells derived from pluripotent stem cells (PSCs), have amazing potential to treat a wide variety of diseases and vast numbers of patients globally. However, there are various challenges related to the manufacturing of PSCs in large enough quantities to meet commercial needs. This manuscript addresses the challenges for the process development of PSCs production in a bioreactor, and also presents a scalable bioreactor technology that can be a possible solution to remove the bottleneck for the large-scale manufacturing of high-quality therapeutic cells derived from PSCs.

## 1. Introduction

With their potential to cure a wide variety of disease indications and address vast patient populations, allogeneic cell therapies derived from pluripotent stem cells (PSCs) are poised to revolutionize therapeutic medicines [1,2]. However, 2D planar manufacturing technologies that have been commonly used for small scale R&D and early stage clinical trials are inadequate and cost-prohibitive for production at the larger scales required for late-stage clinical trials and commercial manufacturing [3]. Single-use bioreactors are widely recognized as a feasible manufacturing solution but need to be optimized in order to meet the unique process requirements of PSCs [4]. One of the critical challenges for future success in commercializing allogeneic cell therapy products is establishing a scalable manufacturing technology that can reliably reproduce the yield and quality of PSC-derived products generated from small-scale R&D at larger scales sufficient for commercial manufacturing [5].

PSCs are mortal human cells that include specific cell types such as human embryonic stem cells (hESCs) and induced pluripotent stem cells (iPSCs). When cultivated in a 2D planar vessel, PSCs attach to a surface substrate and grow as a monolayer. In contrast, starting from single cells or small clumps in suspension bioreactors, PSCs that come into contact will naturally clump together to form spherical cell aggregates [6]. The formation of cell aggregates is required not only for the cell expansion phase but also for subsequent differentiation, which can be a multi-step process that directs the pluripotent cells to turn into a final target cell type for treating a particular disease.

Depending on a particular cell expansion and differentiation process, there is an optimal range of spherical cell aggregate sizes that can maximize the efficiency and production yield of expansion and differentiation processes in a bioreactor [7,8,9]. If a PSC aggregate becomes too large, nutrients and the growth or differentiation factors may be unable to evenly diffuse from the aggregate surface into its center, leading to unwanted cell death or heterogeneous cell populations during expansion or differentiation [10,11]. PSC aggregates that are too small may result in less efficient cell expansion and differentiation, thus lowering the yields of final target cells [12].

There are varying published examples of optimal cell aggregate sizes for different cell types and cell culture process steps. One experiment using hESCs showed that maximum viability and minimal cell apoptosis during expansion was achieved when average aggregate diameter was 300 μm after 7 days of expansion, while apoptosis (with the majority of necrotic cells in the centers of aggregates) peaked by day 14 if the average diameter reached 500 μm [13]. In another experiment, hESC aggregates with diameters of 400 μm detrimentally had half the concentration of oxygen in their centers compared to aggregates of 200 μm [10]. In a different experiment focusing on hESC differentiation, an average diameter of 450 μm was ideal for cardiogenesis, whereas diameters between 150–300 μm were ideal for endothelial cell differentiation [14]. For two different experiments involving hPSCs, an average aggregate diameter of 139 ± 26 μm was optimal for initiating differentiation into neural cells in one experiment [15], while an average diameter of 130 ± 40 μm was optimal for endoderm induction in the other experiment [16]. As these various experiments indicate, optimal cell aggregate size is highly dependent on factors such as cell type and expansion or differentiation process steps.

While PSC aggregate formation can be influenced by variables such as cell proliferation rate, cell–cell adhesion strength, and cell packing density, the hydrodynamic environment inside a bioreactor, which is created by the impeller used to continually mix the liquid media, also has a significant impact on determining aggregate size and, ultimately, cell viability [17]. The hydrodynamic environment is characterized by the two parameters of fluid shear stress and turbulent energy dissipation rate. Both of these parameters are inversely correlated with the average size of PSC aggregates: higher levels of shear stress and turbulent energy dissipation rate result in smaller sized aggregates, and vice versa. After the initial seeding of single cells or small preformed aggregates into a bioreactor, collisions due to the hydrodynamic environment will facilitate aggregate growth, either through the addition of single cells onto existing aggregates or the fusion of smaller aggregates into larger ones. At sufficiently higher agitation rates, the increased levels of shear stress will promote the breakage of loosely attached or temporarily agglomerated larger aggregates and thus limit their maximum possible size [18]. 

Fluid mixing in a bioreactor using a traditional horizontal-blade impeller creates a significantly uneven hydrodynamic environment. The highest levels of shear stress and turbulent energy dissipation rates will be near the tips of the rapidly spinning impeller, with decreasing gradients of these hydrodynamic parameters as the distance from the impeller increases [19,20]. Such a wide range of shear stress levels and energy dissipation rates in a horizontal-blade impeller bioreactor results in a non-uniform hydrodynamic environment, which in turn results in a wide variation in PSC aggregate sizes. In this scenario the size of cell aggregates will vary and the range of aggregate diameters in suspension becomes broad, which ultimately results in an inconsistent yield and quality of PSCs during expansion and differentiation steps. This variation in PSC aggregate size becomes more pronounced at larger sizes of horizontal-blade impeller bioreactors. 

## 2. Scalable Bioreactor Technology as Manufacturing Solution

**Figure 1:**
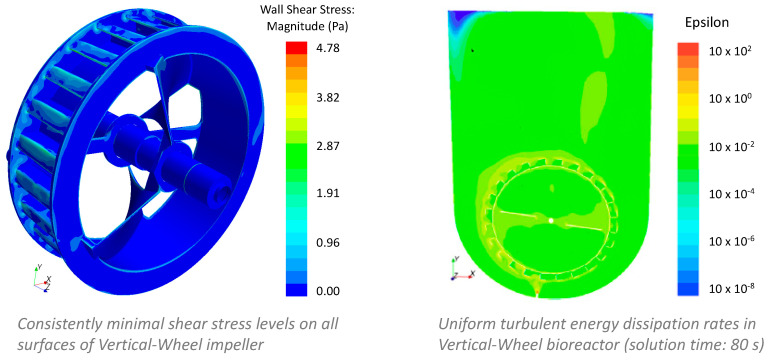
Computational fluid dynamics (CFD) analyses of shear stress on the surface of vertical-wheel impeller and range of turbulent energy dissipation rates in U-shaped vessel.

In contrast, the innovative Vertical-Wheel™ impeller, in conjunction with a distinct U-shaped vessel, provides a significantly more homogeneous hydrodynamic environment in Vertical-Wheel bioreactors. Computational fluid dynamics (CFD) analysis shows consistently low shear stress levels on all surfaces of the Vertical-Wheel impeller, as well as a narrow range distribution of turbulent energy dissipation rates throughout the U-shaped vessel (Figure 1) [21].

**Figure 2:**
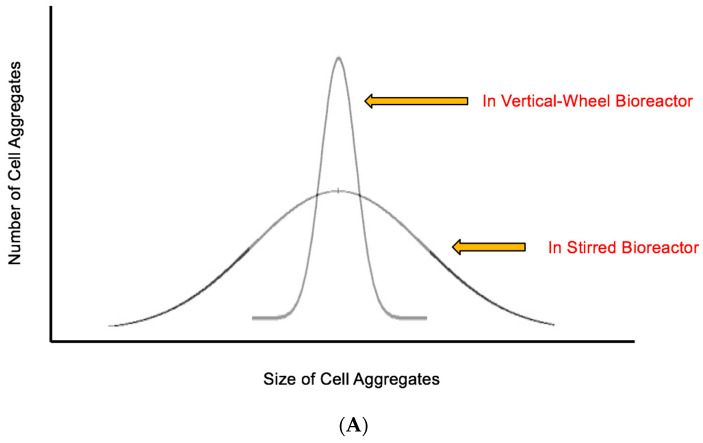

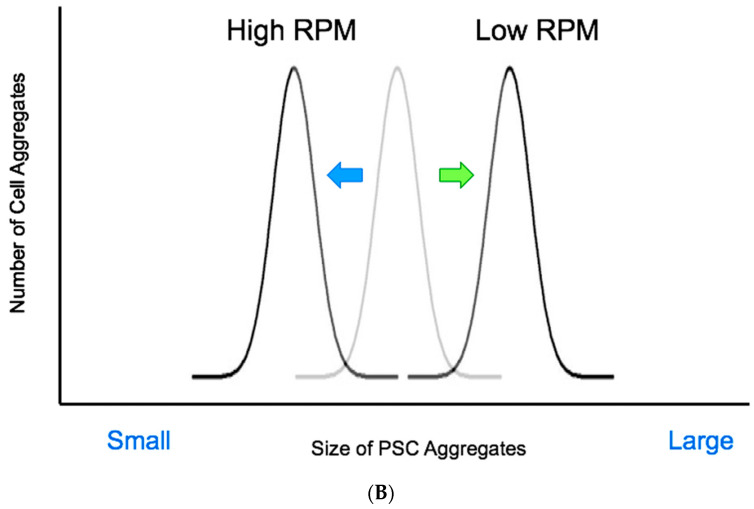
(**A**) Projected distribution of cell aggregate sizes in Vertical-Wheel bioreactor (homogeneous) vs. horizontally stirred bioreactor (non-homogeneous) hydrodynamic environments. (**B**) inverse correlation between average pluripotent stem cells (PSC) aggregate diameter and agitation rate in Vertical-Wheel bioreactors.

Fluid mixing using the Vertical-Wheel results in a very low variation in these two hydrodynamic conditions while still achieving the complete and continual suspension of PSC aggregates. The homogeneous hydrodynamic environment will result in a much tighter distribution of PSC aggregate size (Figure 2A). As a result, PSC aggregates in a Vertical-Wheel bioreactor will consistently have spherical shapes with similar diameters. 

In addition, the average diameter of PSC aggregates can be controlled by simply adjusting the agitation rate of the Vertical-Wheel impeller (Figure 2B). As agitation rate increases, turbulent energy dissipation rates and shear stress levels also increase while maintaining a homogeneous hydrodynamic environment, resulting in smaller average aggregate diameters and still narrow size distributions. The inverse is also true, with average aggregate sizes becoming larger as agitation rate is lowered. Starting with a relatively low agitation rate after seeding can promote initial aggregate spheroid formation. The agitation rate could later be increased to a speed that completely suspends larger particles and forms optimally sized cell aggregates, while also preventing unwanted fusion or agglomeration. Furthermore, the homogeneous hydrodynamic environment is maintained as bioreactor volume increases. Being able to predict the mixing properties and hydrodynamic environment at larger scales based on process development done at a small scale will be enormously beneficial for establishing a scalable manufacturing process for PSCs.

## 3. Independent Biological Performance Data from Collaborators

**Figure 3:**
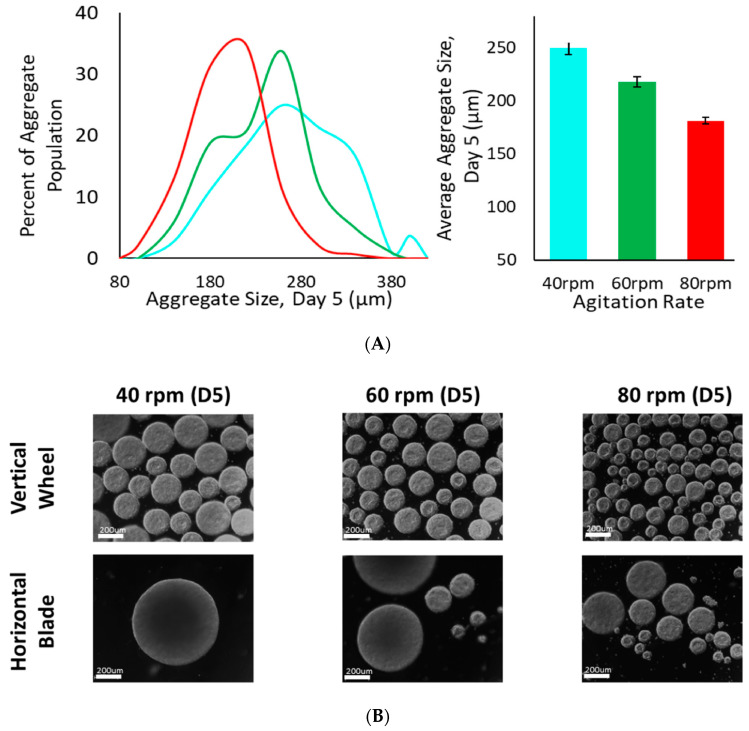
(**A**) Inverse correlation between average iPSC aggregate diameter and agitation rates in Vertical-Wheel bioreactor (0.1 L Scale). (**B**) Comparison of iPSC aggregate diameters and morphology with different agitation rates in Vertical-Wheel bioreactor vs. horizontal-blade spinner (0.1 L scale). Scale bar (200 µm).

Biological studies in Vertical-Wheel bioreactors have confirmed the inverse correlation between agitation rate and average size for iPSC aggregates (Figure 3A). The uniformity of cell aggregate sizes at different agitation rates in small-scale Vertical-Wheel bioreactors, compared to those produced in horizontal spinner flasks, was also confirmed (Figure 3B) [22]. The observed narrow size distributions and uniform morphology also indicate that unwanted agglomeration into overly large or irregular spheroids is minimized across various agitation rates.

**Figure 4:**
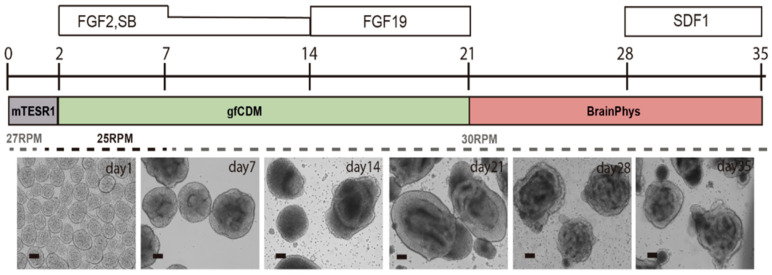
Directed differentiation of human iPSCs into cerebellar organoids in 0.1 L scale Vertical-Wheel bioreactor. Scale bar (100 µm).

As previously mentioned, the uniformity of PSC aggregates is essential not only for the efficiency of cell expansion, but also for directed differentiation, by minimizing the chance that growth and differentiation factors diffuse unevenly through aggregates, and therefore avoiding the undesired heterogeneity and lower quality of target cells. In another set of experiments, iPSC aggregates were successfully differentiated into neural cells, which then formed cerebellar organoids in suspension within small scale Vertical-Wheel bioreactors (Figure 4). After 35 days of a cell expansion and differentiation process, iPSC-derived organoids were efficiently maturated to GABAergic and Glutamatergic neurons in 0.1 L scale Vertical-Wheel bioreactors [23]. Other iPSC aggregates were successfully differentiated into beta cells, cardiomyocytes, or mammary organoids in suspension using different differentiation processes in various scales of Vertical-Wheel bioreactors (data not shown). In addition, studies to differentiate iPSCs into liver organoids in suspension are currently ongoing.

While these various experiments were performed at relatively small scales (0.1 to 15 L) in Vertical-Wheel bioreactors, the homogeneous hydrodynamic environment created by the Vertical-Wheel impeller and U-shaped vessel has been modelled by CFD analysis up to 80 L scale and beyond. Therefore, it can be predicted that the formation of uniformly sized PSC aggregates will also be scalable in Vertical-Wheel bioreactors, resulting in efficient cell expansion and differentiation processes even at larger scales.

**Figure 5:**
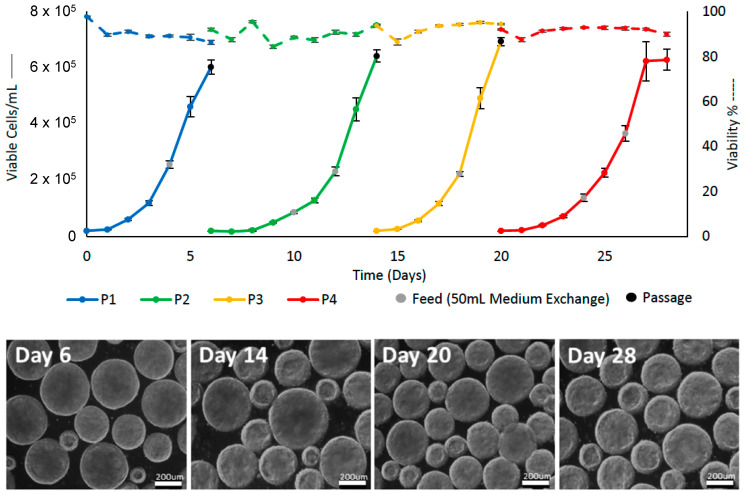
Successful Serial Passaging of iPSCs in 0.1 L Scale Vertical-Wheel Bioreactors.

A PSC expansion process can start with a small cryopreserved vial of PSCs from a working cell bank, inoculating thawed cells into a small-scale bioreactor, and then expanding the cells by serially passaging them into progressively larger bioreactors. Using the scalable bioreactor system with a consistent hydrodynamic environment will improve the efficiency of serial passaging and total cell yield. Studies were performed in order to determine the consistency of PSC expansion in Vertical-Wheel bioreactors, from small-seed culture scale (0.1 L) to potential production scale (80 L). Freshly thawed iPSCs were inoculated into a 0.1 L Vertical-Wheel bioreactor and then four consecutive passages of iPSCs were performed using scale-down model bioreactors of identical size. These studies repeatedly demonstrated that the use of Vertical-Wheel bioreactors led to an average 32-fold expansion of iPSCs during 6–8 days of culture period per passage, achieving a cumulative cell expansion of greater than one million-folds in 28 days (Figure 5). The cells harvested at the end of the serial passage were of high quality, maintaining a normal karyotype, pluripotency, and the ability to form teratomas in vivo. This scale-down model process suggests that commercial scale production of over one trillion high quality iPSCs could feasibly be generated in a Vertical-Wheel bioreactor with 50 L working volume [22]. 

Various scaling factors can be used to the determine potential scalability of expansion and differentiation processes developed at a small scale in Vertical-Wheel bioreactors. Common parameters that would need to be kept constant during scale-up include Reynolds number, impeller tip speed, power input, maximum shear stress, velocity, and energy dissipation rate. A recent study has suggested that maintaining the volume average energy dissipation rate, determined through computational fluid dynamics simulations, is the best scale-up method for predicting the agitation rates that will sustain a desired average aggregate diameter with narrow size distribution [24]. Therefore, the agitation rates for each progressively larger Vertical-Wheel bioreactor can be calculated that will maintain the same homogenous hydrodynamic environment that was determined during process development in small-scale Vertical-Wheel bioreactors.

## 4. Conclusions

The ultimate goal of providing allogeneic PSC-derived products reliably to vast numbers of patients requires a series of optimized unit operations at various scales to meet target manufacturing lot sizes. Numerous manufacturing processes need to be considered, including seed train, expansion, differentiation, cell harvest, wash, concentration, fill-finish, and cryopreservation. In particular, upstream cell culture processes represent the most challenging bottleneck to achieving robust and economical manufacturing at larger scales. With homogeneous hydrodynamic environments and unparalleled scalability, Vertical-Wheel bioreactors can serve as the innovative manufacturing platforms to enable commercial-scale production of PSC-derived therapeutic cells and to secure the supply of these novel therapeutic cell products to patients.
